# Study on the Application of Sediment-Based Embankment Building and Ultra-High-Performance Concrete (UHPC) Preparation in the Resource Utilization of Yellow River Sediment

**DOI:** 10.3390/ma15165668

**Published:** 2022-08-18

**Authors:** Jun Yan, Sai Zhong, Sainan Chen, Yajun Lv, Longbin Yang, Guanghong Peng, Anjun Deng

**Affiliations:** 1 School of Water Conservancy, North China University of Water Resources and Electric Power, Zhengzhou 450046, China; 2 Wuhan Electric Power Vocational and Technical College, Wuhan 430072, China; 3 State Key Laboratory of Simulation and Regulation of Water Cycle in River Basin, China Institute of Water Resources and Hydropower Research, Beijing 100046, China

**Keywords:** sediment, resource utilization, sediment-based embankment building, ultra-high-performance concrete test

## Abstract

The Yellow River is difficult to control. Little water and a large amount of sediment results in sediment accumulation in its lower reaches as sediment inflow exceeds transport capacity. Reducing this sediment deposition is essential for harnessing the Yellow River. Included in this process is the rational use of the sediment. Many researchers have investigated usage of Yellow River sediment as an aggregate material for concrete production, but there are still some problems (e.g., low resource utilization and low strength of the concrete made from Yellow River sediment). To make up the deficiency in the existing research, this study proposes two methods of sediment utilization. One is to use Yellow River sediment to build embankments, and the other is to use ultra-fine Yellow River sand to prepare ultra-high-performance concrete (UHPC). Test results reveal that the prepared high-strength concrete performs well in each test, including: fluidity, mechanical properties, pore structure, ecological evaluation, microscopic measurement of the interface transition zone, and economic analysis.

## 1. Introduction

The Yellow River is one of the most difficult rivers to harness worldwide, as it is characterized by complicated hydrological and sediment conditions [1]. Since the founding of the People’s Republic of China, the State has invested considerable human [2], material, and financial resources in the control of Yellow River sediment. Since the 1950s, China has progressively proposed and optimized measures of “blocking, discharging, regulating and digging” for sediment control, and has made some progress. In the 21st century, the previous “blocking, discharging, releasing, regulating and excavation” was modified to “blocking, discharging, regulating, releasing and excavation” + “use”, and clearly proposes the “resource utilization of sediment”. Sediment resource utilization [3] has been considered one of the directions to solve the sediment problem of the Yellow River. “Resource utilization” is the utilization or recycling of sediment as raw materials, and sediment resource utilization has great economic and ecological benefits. Several years of sediment resource utilization have achieved good results; however, there are also some problems (e.g., the low amount of sediment and the low strength of concrete made from Yellow River sand).

Thus, this study proposes two sediment resource utilization methods to address the current shortcomings: using the deposited sediment in the reservoir area to construct an embankment to improve the storage capacity, and using the ultra-fine sand of the Yellow River to prepare ultra-high-performance concrete (UHPC). The particle size of ultra-fine Yellow River sand in the Zhengzhou section is similar to that of small quartz sand (0.18~0.6 mm), and the particle size of cement (<0.075 mm) is similar to that of Yellow River silt in the Puyang section. Therefore, it is feasible to use mixed sand composed of Yellow River ultrafine sand (0~0.6 mm) and river sand instead of quartz sand as the fine aggregate in high-performance concrete, and to use Yellow River silt (0~0.072 mm) instead of cement to prepare high-performance concrete. This provides a new source for concrete building materials and allows resource utilization of the sediment of the Yellow River, which will bring good economic and ecological benefits to the lower reaches of the Yellow River.

## 2. Previous Work

From flood diversion, desilting, mechanical desilting, and embankment consolidation since the last century, to sediment brickmaking and soil improvement over the past few years, decades of exploration has resulted in great progress in the utilization technology of sediment in the Yellow River. 

### 2.1. Sediment Used for Land Rectification

Sediment used for land rectification can be divided into flood diversion and desilting, and sediment filling of collapsed mining areas [4,5]. To be specific, common flood diversion and desilting technologies comprise ditch dredging, village platform silting, and farmland improvement [6,7,8].

**Figure 1:**
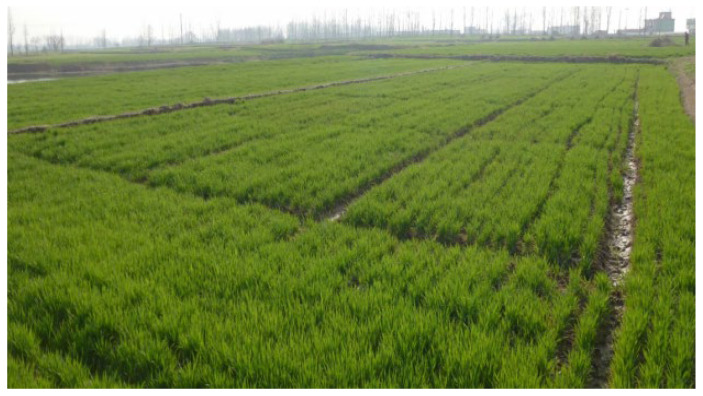
Farmland improvement.

(1) Flood diversion and desilting: The silted Yellow River sediment is adopted to fill dike ditches and cascade ditches on the beach surface to improve the impermeability of the dike and to prevent the river from rushing directly into the dike along the cascade ditch. Based on silting treatment, the probability of flood discharge along the embankment and the occurrence of cross river, oblique river, and rolling river decreases, thus ensuring flood-control safety of the Yellow River embankment to a certain extent. First, the sediment of the Yellow River can consume the sediment deposited by the Yellow River. Further, the construction of village platforms and centralized resettlement can save considerable land resources and improve the carrying capacity of land resources for economic and social development.
(2) Sediment filling of collapsed mining areas: There are a considerable number of coal mines in the Yellow River Basin [9]. Out of the 26 large coal fields in China with proven reserves of more than 10 billion tons, 12 are in the Yellow River Basin. Due to the shallow burial of coal seams and unfavorable construction, numerous coal mining subsidence areas have been formed. Shandong alone has five areas: Jining Zaozhuang Plain Lake, Tai’an Laiwu Hills, Jinan Dezhou Binhuang River, Heze Plain, and Longkou Binhai Plain. First, coal mines can save subsidence land compensation and cultivated land occupation tax every year due to safe production and mine standardization construction, which reduces the economic burden of enterprises. Further, this improves agricultural conditions by enhancing drought and flood resistance, land productivity, and output.
(3) Farmland improvement: Using the sediment of the Yellow River to improve farmland [10] can significantly enhance soil physical properties (Figure 1), reducing soil bulk density, increasing porosity, facilitating effective infiltration and precipitation storage, reducing surface runoff, and preventing water and soil loss.

### 2.2. Sediment Use in Construction Industry

Sediment use in the construction industry includes making bricks and ceramsite [11,12,13].

(1) Sand brickmaking. With China’s increasing attention to the ecological environment, sources of building materials that have an impact on the environment, such as mountain quarries, are limited, and Yellow River sediment brickmaking and flood control stone materials have progressively aroused the attention of society. Boxing County [14] of Shandong province has successfully trail-produced new wall materials with Yellow River sludge as a raw material over the past five years. The products comprise hollow bricks, perforated bricks, decorative bricks, and others, which are characterized by their high strength, thermal insulation, energy savings, and no radioactivity. The project has been covered in the first batch of key projects of the “double high and one excellent” guiding plan by the state.

While meeting national environmental protection requirements, sediment brick also shows several advantages, including fire resistance, weight reduction, earthquake resistance, shear resistance, and energy savings. Preparation of flood-control stones from the sediment of the Yellow River can alleviate the flood-control pressure in its lower reaches while satisfying economic development needs along the river and facilitating the development and progress of sediment resource utilization technology and equipment.

(2) Sand ceramsite: Yellow River sediment can be excavated, naturally dried, pelletized, preheated, roasted, and cooled into ceramsite. As a result, sediment resource utilization has been achieved. Using the sediment of the Yellow River to replace clay to make pottery [15] reduces the use of clay, ceramsite, and other filler materials, protecting clay and other resources.

### 2.3. Preparation of Concrete from Yellow River Sand

In order to alleviate the shortage of medium-coarse sand [16], Yellow River ultra-fine sand is used to partially replace medium-coarse sand to prepare a Yellow River sand concrete test block. Test results show that the addition of Yellow River ultra-fine sand improves the gradation, mechanical properties, pore structure, impermeability, and frost resistance of the concrete, and the workability of the concrete meets the pumping requirements of commercial concrete.

**Figure 2:**
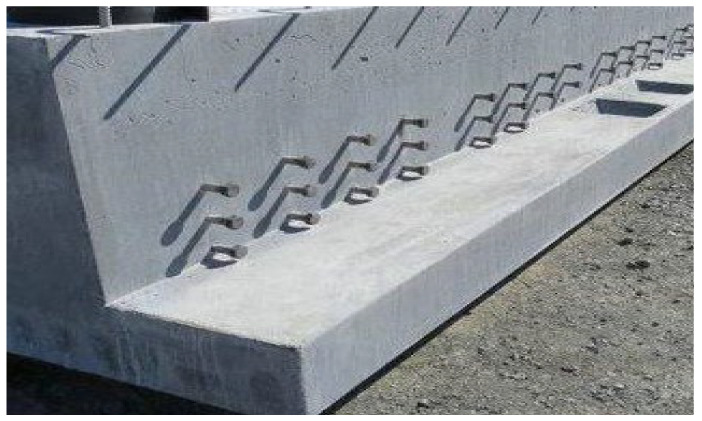
Ultra high-performance concrete (UHPC).

Test results show that the mechanical properties of the samples are related to the curing conditions [17]. The mechanical properties of the samples under standard curing are higher, and their strength increases with increased curing time. In addition, the content of Yellow River ultra-fine sand also affects mechanical properties. When the content of Yellow River ultra-fine sand is 15% or 30%, the mechanical properties of the concrete are good, and early shrinkage performance of the concrete is best when the content is 15%. (Figure 2).

The concrete mix proportions of different strength grades are obtained through testing [18]. Ultra-fine sand of the Yellow River is mixed into machine-made sand in proportions of 10%, 20%, 30%, 40%, and 50% to prepare a test block. The test results show that the sand obtained by mixing ultra-fine sand of the Yellow River and artificial machine-made sand in a certain proportion can greatly improve the gradation between particles. The flowability of concrete also increases with increased content of superfine Yellow River sand. For C20, C30, C40, C50, and C60 grade concrete, the best content is 20%, 30%, 30%, 30%, and 20% respectively.

In order to study the influence of different contents of Yellow River sand on different grades of concrete [19], concrete test blocks are prepared by partially replacing machine-made sand with Yellow River sand. The test results show improved working performance of the Yellow River sand concrete mixture. In addition, the mechanical properties are generally improved. However, the amount of Yellow River sand has different effects on different grades of concrete, with more Yellow River sand able to be incorporated in low-strength concrete than in high-strength concrete.

By changing the ratio of superfine Yellow River sand to river sand [20], as well as the water–cement ratio and water–binder ratio, the permeability of plastic concrete is studied. The results show that superfine Yellow River sand and river sand mixed in a certain proportion can be used as the aggregate for preparing plastic concrete. The optimal ratio of 50% uses less cement and has good impermeability.

Taking Yellow River sediment as a raw material, flood control stone is prepared by alkali activation [21], and then the effects of alkali activator content, slag content, and curing age on the mechanical properties are studied. The test shows that the best mix proportion is when the slag content is 10%, ${\mathrm{Ca}}_{2}$ (OH) content is 5%, and NaOH content is 0.5%. In addition, sem/eds, tg/dtg, and other tests show that the main product of alkali activation is hydrated calcium silicate gel.

Regarding sediment control in the Yellow River, the above sediment resource utilization scheme has studied sediment utilization of the Yellow River from different perspectives and has achieved certain results (e.g., increasing the land utilization rate of the beach area, improving the soil of the beach area, optimizing sediment brickmaking technology, and protecting clay resources). However, there have also been the following deficiencies: (1) After decades of sediment resource utilization, it has played a certain role in reducing river sedimentation. At present, however, the exploited sediment only accounts for a small part of the deposited sediment, and the sediment utilization rate is small. (2) Although different researchers have tried to optimize temperature, pressure, and dosage to for Yellow River sand concrete, the strength of the concrete is generally not high.

Thus, to solve the first shortcoming, this study proposes sediment-based embankment building on the Yellow River, which transports the sediment from the bottom of the reservoir to the water’s surface and uses the transported sediment to build the embankment; this will consume considerable sediment and improve the sediment utilization rate. In view of the second deficiency, we propose preparing high-strength concrete to solve the low strength of Yellow River sand concrete.

## 3. Materials and Methods

### 3.1. Sediment-Based Embankment Building

Sediment-based embankment building is the transportation of sediment from low to high. The target is the considerable amount of sediment deposited in the Sanmenxia Reservoir area. The cleared sediment is treated on-site and built into a dike at the elevation line of 330 m on both banks of the reservoir area.

#### 3.1.1. Feasibility Study

**Figure 3:**
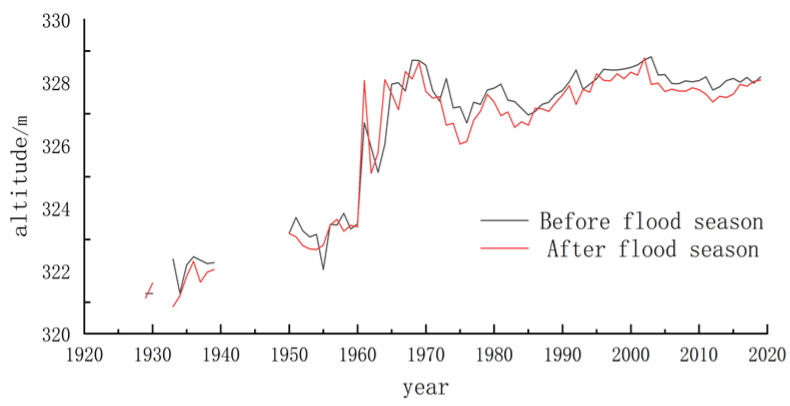
Annual average elevation change of water surface in Sanmenxia Reservoir area.

In 1960, Sanmenxia Reservoir [22,23,24,25] was put into operation. September 1960 to March 1962 was a period of water storage and sediment detention (Figure 3). The highest pool level reached 332.58 m in 1961.2, and the reservoir water level was above 330 m for 200 days. From June 1962 to June 1964, it was maintained at 325.11 m to 326.02 m. In 1964, it rose to 328.09 m after the flood season in a year of abundant water and sand. It was reconstructed initially in July 1966 and dropped to 327.13 m after the flood season. It was 328.55 m at the beginning of the flood season in 1970. At the end of the 1973 flood season, it was 326.64 m, 3.24 m higher than before the construction of the reservoir.

The period since November 1973 has consisted of storing clear water and discharging muddy water. In the non-flood season, the highest operating water level of the reservoir has decreased yearly, and the duration of the high water level has also decreased yearly. From 1974 to 1979, the reservoir water level was higher than 322 m an average of 74 days annually. From 1979 to 1985, the reservoir water level exceeded 322 m for an average of 57 days. From 1986 to 1992, the reservoir water level reached over 322 m an average of 39 days annually. From 1993 to 2001, the reservoir water level exceeded 322 m for an average of 3 days. In 2003, “318” was put into operation [26], and the operating water level in the non-flood season does not exceed 318 m. At present, the water level in the non-flood season of Sanmenxia is controlled at 321 m, and Q > 2500 m^3^/S in the flood season; otherwise, it is limited to 305 m.

As revealed by the above data, the historical pool level of the Sanmenxia Reservoir area is more than 330 m part of the time and more than 322 m for a large proportion of time. Accordingly, it is feasible to build an embankment on the 330 m elevation line on both banks to restore the water level in the reservoir area to 330 m.

#### 3.1.2. Embankment Scheme Planning

**Figure 4:**
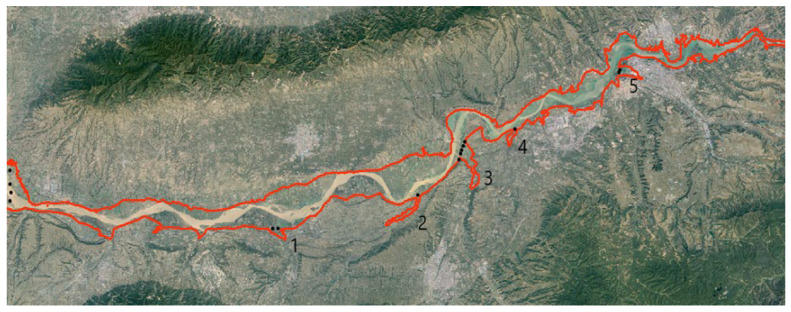
The 330 m elevation line of Sanmenxia Reservoir area.

An elevation line of 330 m is drawn from Tongguan to the dam in the reservoir area (Figure 4), the sediment deposited in the reservoir is dredged and sent to the bank, and a dike is built at an elevation of 330 m on both banks of the river. The geometric dimensions of the embankment section are 1.5–2 times that of a standardized embankment [27,28] in the lower reaches of the Yellow River. The height of a standardized embankment in the lower reaches of the Yellow River is nearly 10 m, the top width is 12 M, and the slope ratio of the embankment adjacent to and back to the river is 1:3. Thus, the embankment in the reservoir area is developed with an upper bottom width of 24 m, a lower bottom width of 114 m, and a height of 15 m.

**Figure 5:**
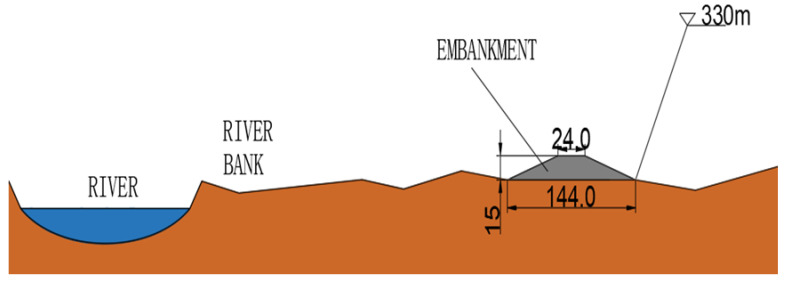
Vertical transport reservoir section.

Under the restraint that the living conditions of residents remain unchanged, we take the bottom of the embankment as a horizontal line and extend it 10–20 m to the outside (the broken line in Figure 5) and fill it with sediment between the embankment and the extension line to improve the strength and seepage resistance of the embankment. 

**Figure 6:**
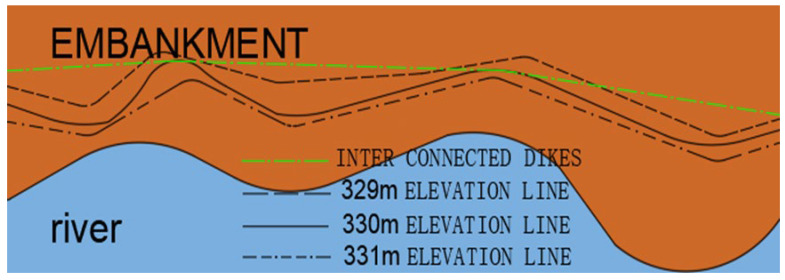
Embankment building scheme (north bank).

Due to the terrain, the 330 m elevation line is not straight. Notably, V-shaped elevation lines often form in gullies. Accordingly, embankment building adopts an internal connection scheme. Internal connection is the interconnection of some vertices at the 330 m elevation line on the side close to the river beach. The scheme adopts straight embankments (Figure 6). This can leave a large number of beach areas, thus contributing to safe flood discharge and the construction of Swan Lake wetland. Completing the embankment will form a triangular gully zone (1, 2, 3, 4, and 5 in Figure 4). The gully zone has sufficient water and flat terrain for the construction of Swan Lake wetland.

#### 3.1.3. Embankment Cost

(1) Sediment extraction cost: The total cost of sand excavation consists of sand dredger rental, power, labor, and oil. Dredger rental would cost from 80 million yuan to 120 million yuan, which is equivalent to the purchase price. The salary of the personnel over the total building period would be nearly 60–70 million yuan plus other expenses (e.g., electricity, oil, and ship loss). The sediment extraction cost would be about 200–300 million yuan.
(2) Building of embankment and relocation cost: The cost of dump trucks and excavators would be about 160 million yuan. Oil, electricity, and other related expenses are expected to cost between 20–30 million yuan. The total transportation cost ranges from 200 to 300 million yuan. The cost of concrete would be approximately 80–90 million yuan. The labor cost and the excavator cost would reach nearly 130 million yuan. Compensation for resident relocation is based on a population of approximately 50 thousand people in the Sanmenxia Reservoir area, and the cost would range from 4 to 4.5 billion yuan.

#### 3.1.4. Engineering Benefit

Sediment-based embankment building achieves on-site utilization of sediment while bringing the following benefits:

(1) Dredging: Building sediment revetments significantly reduces the amount of sediment deposition in the reservoir area, enhances the water storage performance of the reservoir, and utilizes rather than wastes the cleared sediment.
(2) Swan protection: Swans are second-class protected animals. Tens of thousands of white swans migrate from Siberia to Sanmenxia for winter every October. The construction of Swan Lake wetland community will provide tens of thousands of mu of habitat for swans.
(3) After the water surface in the Sanmenxia Reservoir is raised, alkali pressing can be achieved, and farming conditions, soil fertility and quality, and crop yield can be improved, increasing agricultural income.

### 3.2. Ultra-High-Performance Concrete Made of Yellow River Sand

#### 3.2.1. Raw Materials

The materials selected for the preparation of ultra-high-performance concrete (UHPC) consist of ordinary Portland cement, green high-performance polycarboxylic acid superplasticizer (solid content reaching 30%, water reduction rate of 30%), first-class fly ash, silica fume, and fine aggregates, which consist of large-size river sand (0.6~1.18 mm), quartz sand (0.18~1 mm), and ultra-fine Yellow River sand (0~0.6 mm). Table 1 lists the composition of raw materials as obtained by XRD.

#### 3.2.2. Mix Proportion Design

##### Design Method

High-strength concrete benefits from its dense skeleton stacking structure. A dense internal structure gives mechanical properties and pore microstructure density several times that of ordinary concrete [29]. Thus, this study uses the modified A&A model (Andreasen and Andersen [30] proposed a continuous particle-accumulation model: through modification and improvement, the particle size of all solid materials can be optimized, and the proportion of each material can be determined according to the optimized target curve) to design the mix proportion of ultra-high-performance and high-strength concrete, using Equation (1) [31] to regulate the proportions of various ingredients to obtain the optimal mix to ensure a dense stacking structure.

(1) $$P\left(D\right)=\frac{D^{q}-D_{\min}^{q}}{D_{\max}^{q}-D_{\min}^{q}}$$

where D is particle size (µm), P(D) is part of the total particle size (with particle size ranges less than D), $D_{\max}$ is the largest particle size (µm), $D_{\min}$ is the minimum particle size (µm), and q is the distribution modulus (taken as 0.23).

Based on the modified A&A model, the mix proportion of concrete can be designed. The proportion of various materials in concrete is of great significance, as it determines the density of the stacking structure of the prepared concrete. Because our research is on ultra-high-performance and high-strength concretes, only fine aggregate is used. The content of fine aggregate is dependent on the distribution modulus, which is fixed at 0.23 in this study. Next, the mass proportion of various materials in the concrete mortar is regulated using the least-square method (LSM) as seen in Equation (2). (The volume fractions of each component are taken as the independent variables and adjusted to minimize the difference between the mix-proportion curve and the objective curve; the criterion for discrimination is the sum of squares of the residuals of the content percentages of variously sized particle in the two curves. The French scientist Legendre independently invented the “least square method” in 1806).

(2) $$\mathrm{RSS}=\frac{\sum_{i=1}^{n}{\left(P_{\mathrm{mix}}\left(D_{i}^{i+1}\right)-P_{\mathrm{tar}}\left(D_{i}^{i+1}\right)\right)}^{2}}{n}$$

where $P_{\mathrm{mix}}$ is the particle size of the concrete mixture, $P_{\mathrm{tar}}$ is the target particle size, and n is the calculated target particle size (between $D_{\min}$ and $D_{\max}$) used to calculate the deviation value.

**Figure 7:**
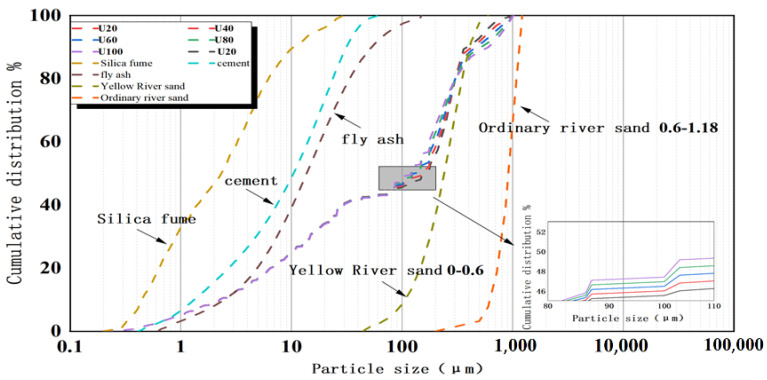
Particle size distribution of raw materials, target curve, and optimization curve of ultra–fine sand mixture of the Yellow River.

The fitting quality of the obtained complete particle-size distribution curve is determined and assessed by the coefficient R^2^ (Equation (3)). (The coefficient of determination is also called the determinate coefficient or the decisive coefficient and refers to the ratio of the sum of regression squares to the sum of total deviation squares in linear regression, and its value is equal to the square of the correlation coefficient). R^2^ provides a correlation value between all the fitted particle size curves and the particle size curve of the target mixture.

(3) $$R^{2}=1-\frac{\sum_{i=1}^{n}{\left(P_{\mathrm{mix}}\left(D_{i}^{i+1}\right)-P_{\mathrm{tar}}\left(D_{i}^{i+1}\right)\right)}^{2}}{\sum_{i=1}^{n}{\left(P_{\mathrm{mix}}(D_{i}^{i+1}-\bar{P_{\mathrm{mix}}}\right)}^{2}}$$

where $\bar{P_{\mathrm{mix}}}=\frac{1}{n}\overset{n}{\underset{i=1}{\sum }}P_{\mathrm{mix}}\left(D_{i}^{i+1}\right)$ is an average value obtained from the overall particle size curve. Lastly, the particle size distribution of raw materials, the target curve, and the optimization curve of the two experiments are shown in Figure 7.

##### Mix Proportion

**Figure 8:**
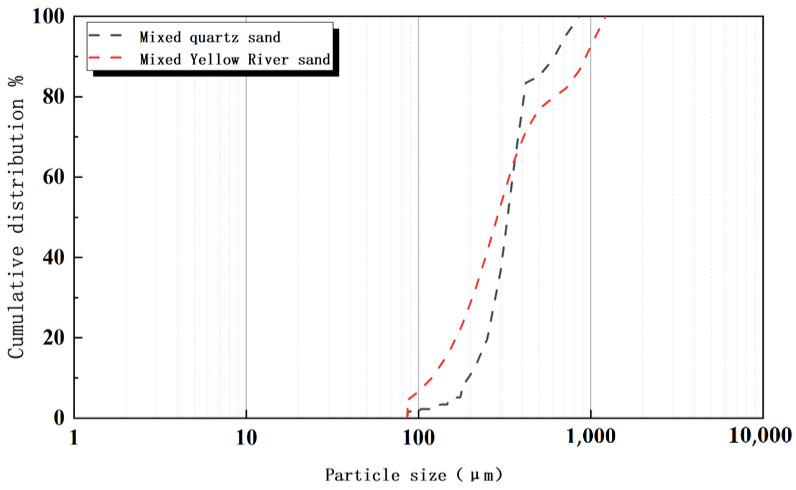
Particle size distribution of mixed sand and quartz sand after mixing.

The particle sizes of the quartz sand used in this test are 0.18~0.6 mm and 0.6~1 mm, while the highest particle size of the ultra-fine Yellow River sand is 0.6 mm(Figure 8). Accordingly, mixed sand composed of 0~0.6 mm ultra-fine Yellow River sand and 0.6~1.18 mm river sand is used to replace the quartz sand at rates of 0%, 20%, 40%, 60%, 80%, and 100% (Table 2). We find that the particle size distribution of quartz sand and mixed sand is relatively similar, which conforms to the principle of approximate replacement of particle size.

#### 3.2.3. Concrete Preparation

First, all the required materials—P · O 52.5 cement (P · O means ordinary Portland cement, and 52.5 means 28-day compressive strength >52.5 MPa), silica fume, fly ash, natural river sand, and other materials—are introduced into a mixing pot and then mixed at 140 + 5 r/min for 2 min. Then 70% of the water and all of the polycarboxylate superplasticizer are added, and mixing continues at 140 + 5 r/min for 2 min. Finally, the remaining water is added to mixing pot, and mixing continues at 280 + 10 r/min for 2 min to obtain the mixture slurry. Then, the slurry is poured into a mold and cures for 24 h at ambient temperature. The sample is then demolded and transferred to a standard curing box for the corresponding curing period. The curing conditions of the curing box are: temperature 20 ± 1 °C and humidity 95%.

#### 3.2.4. Concrete Performance Test

##### Fluidity

**Figure 9:**
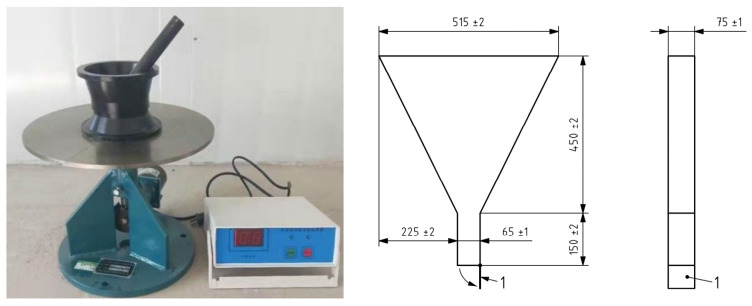
Left: NLD-3 cement sand flow tester; Right: V-funnel.

The GBT2419-2005 standard is used to test the fluidity of concrete mixtures. The instrument used is an NLD-3 cement mortar fluidity tester. The specific test method is as follows: put the mixed mortar into the test mold twice, and tamp it with a tamping rod. After tamping, scrape the excess (the part higher than the test mold) with a knife, start the jumping table, jump 25 times, then lift the cone test mold vertically, and the mixture flows freely. Measure the two mutually perpendicular diameters and calculate their average value (unit: mm), which is the fluidity of the mixture (Figure 9).

When the mixture of river sand and ultra-fine sand of the Yellow River is employed to replace quartz sand at substitution rates of 20%, 40%, 60%, 80%, and 100%, the fluidity is reduced by 0%, 3%, 6%, 12%, and 17%, respectively. This trend is also identified in the V-shaped funnel test. Increased mixed sand content progressively prolongs the time taken to pass through a V-shaped funnel. Replacement rates of 20%, 40%, 60%, 80%, and 100% prolongs the time to pass through a V-shaped funnel by 0.04 s, 0.55 s, 1.26 s, 6.72 s, and 7.20 s, respectively.

**Figure 10:**
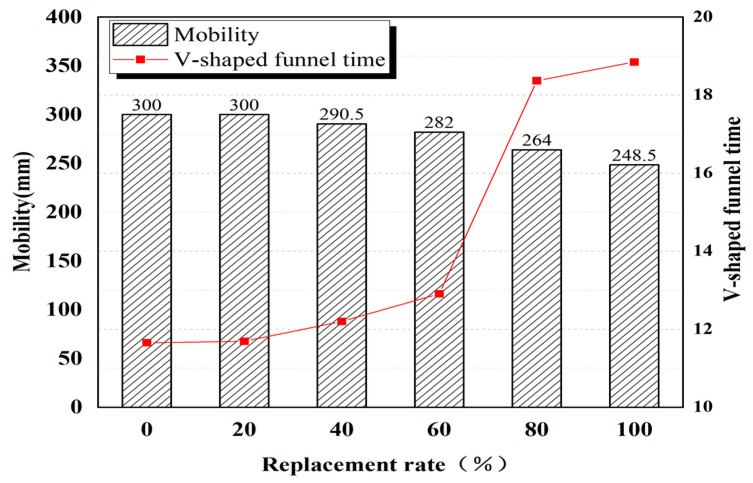
Flow performance of superfine Yellow River sand high-strength concrete.

Nevertheless, even when the replacement rate is 100%, the mixing flow mobility is still 248.5 mm (Figure 10), meeting the requirements of its working performance. The above test results can be attributed to the fact that the ultra-fine sand of the Yellow River is relatively fine and has a larger specific surface area than quartz sand. The fine particles are more likely to absorb water in ultra-high-performance concrete (UHPC), thickening the slurry and decreasing fluidity.

##### Mechanical Properties

**Figure 11:**
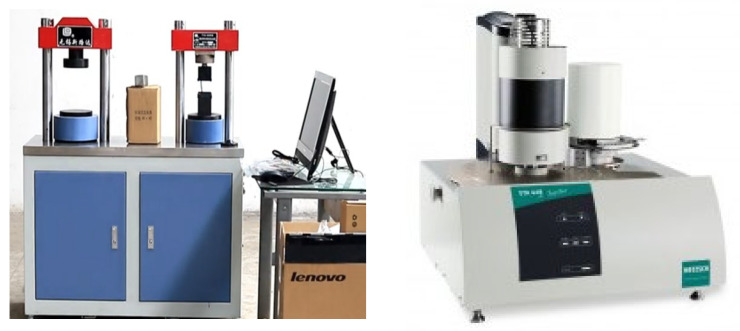
Left: TyA-300B compression and flexometer; Right: STA449F5 synchronous thermal analyzer.

The compressive and flexural strength of the sample is determined in accordance with the standard cement mortar strength test (ISO method) (GB/T 17671-2020), and the instrument used is the fully automatic flexural and compressive testing machine TYA-300B. The specific operations are as follows: first wipe the moisture from the surface of the test block. Then, put the flat side of the test block upward into the bending test machine and start the machine. The loading speed of the bending test is 50 N/s ± 10 N/s until the test block breaks. Average the bending strength results of three samples from each group. The test block for compressive strength is the fractured test block after the bending test. The specific test steps are as follows: put the flat side of the test block upward into the fixture for the compression test. Before the test, ensure that there are no impurities or sand particles on the compression surface or the bottom surface of the fixture, and then start the machine. The loading speed of the compression test is 2400 N/s ± 200 N/s until the test block is crushed. The results are the average compressive strength of six blocks from each group (Figure 11).

**Figure 12:**
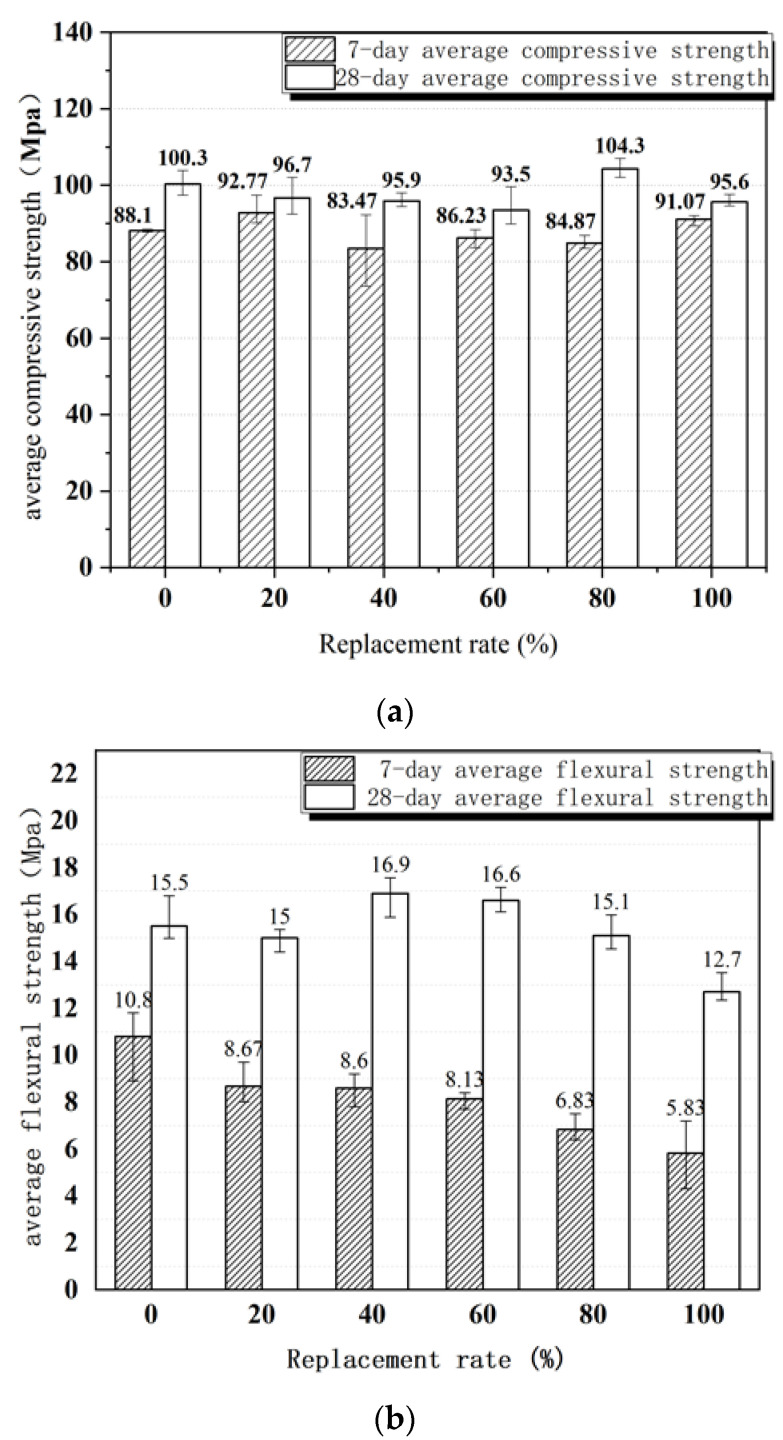
Mechanical properties of high-strength concrete: (**a**) compressive strength; (**b**) flexural strength.

Figure 12 illustrates the compressive strength and flexural strength of ultra-high-performance concrete at 7 and 28 days of age at different mixed sand replacement rates. The compressive strength increases with the increase in the curing period. After 7 days of curing, compressive strengths for replacement rates of mixed sand of 0, 20, 40, 60, 80, and 100% are 88.10, 92.77, 83.47, 86.23, 84.87, and 91.07 MPa, respectively. After 28 days of curing, the compressive strengths increase by 13.8, 4.2, 14.9, 8.4, 22.9, and 5.0%, respectively. The replacement rate of mixed sand has little effect on the compressive strength. When the replacement rates of mixed sand are 20, 40, 60, 80, and 100%, the compressive strengths after 28 days of curing are 96.7, 95.9, 93.5, 104.3, and 95.6 MPa, respectively. There is no significant regularity, whereas the average compressive strength reaches 97.2 MPa.

The trend of flexural strength is similar to that of compressive strength, where the strength increases significantly with the increase in the curing period. When the replacement rates of mixed sand reach 20, 40, 60, 80, and 100%, after 7 days of curing, the flexural strengths are 10.80, 8.67, 8.60, 8.13, 6.83, and 5.83 MPa, respectively. Curing for 28 days enhances flexural strength by 43.5, 73.0, 96.5, 104.2, 121.1, and 117.8%, respectively. This shows that increasing the curing period significantly improves flexural strength. When the replacement rates of mixed sand reach 20, 40, 60, 80, and 100%, the flexural strength at 28 days of curing is 15.5, 15.0, 16.9, 16.6, 15.1, and 12.7 MPa, respectively. Further, the flexural strength does not exhibit a strong regularity, as the general strength is greater than 12 MPa, and the average flexural strength is 15.3 MPa. Notably, with the increase in the replacement rate of mixed sand, the compressive strength and flexural strength do not tend to decrease significantly. The reason for this is that the particle size of Yellow River sand is smaller than that of quartz sand, thus making it more conducive to filling the pores between particles and forming a dense skeleton structure in the concrete.

##### Microscopic Measurement of Interface Transition Zone and Pore Structure

**Figure 13:**
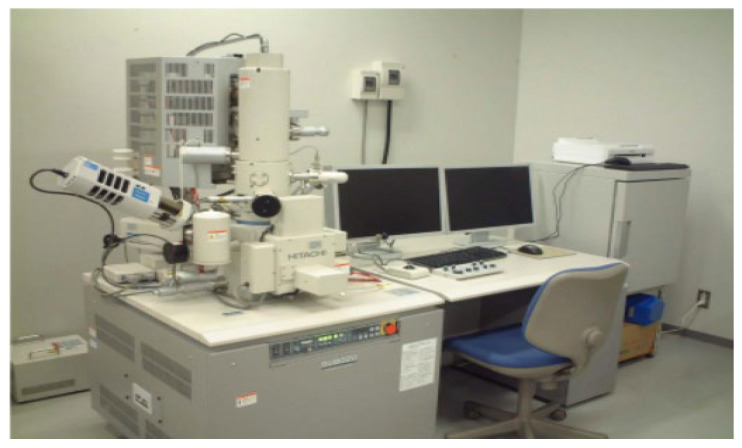
SU8020 emission sweep electron microscope.

The scanning electron microscope used in this test is an emission scanning electron microscope (SU8020) produced by Hitachi company in Tokyo, Japan. Before the test, we put the test block in a vacuum coater and spray a gold film using sputtering to ensure good conductivity (Figure 13).

The interface transition zone is the connection between aggregate and slurry in concrete and is the weakest part of the internal structure. Detailed observation and analysis of the interface transition zone can reveal the overall strength of the sample. The mixed sand comprises 0~0.6 mm ultra-fine Yellow River sand and 0.6~1.18 mm river sand, so the sizes of the aggregates are different. However, in the interface transition zone, the aggregate and slurry are closely connected, and no cracks or pores are found, indicating that the addition of mixed sand will not weaken the interface transition zone of the sample. This again proves that the addition of mixed sand does not seriously reduce compressive strength.

Pore structure is tested using a micro mercury porosimeter (MIP, autopore iv-9500) produced in the produced in Norcross, GA, USA. The specific operation steps are as follows: first, immerse the sample to be tested in isopropanol for 7 d, terminate hydration, vacuum dry for 48 h, and then test.

At 7 days, the cumulative void ratio of all samples mixed with a variety of sands is higher than that of samples without mixed sand. This is due to the relatively small particle size of the ultra-fine sand of the Yellow River, which leads to relatively low fineness and high specific surface area. Thus, it absorbs more free water in the early hydration process [32,33], resulting in faster hydration speed and fewer hydration products. The hydration products cannot fully fill the tiny internal pores, resulting in increased pore proportion. The pore size distribution of all UHPCs is largely concentrated at 20 nm. Increasing the mixed sand replacement rate progressively increases the proportion of pores smaller than 20 nm and decreases the proportion of pores larger than 20 nm. Relevant research suggests that pores smaller than 20 nm are harmless. Pores 20~50 nm are minimally harmful. Harmful pores have diameters of 50~200 nm. If the pore diameter is higher than 200 nm, it is multi-harmful [34,35,36]. After 28 days of curing, the cumulative pore proportion of the sample without mixed sand is no longer the lowest; it is not much different from that of other groups. This is because the ultra-fine sand of the Yellow River is inert and does not react with water. With the increase in the curing period, this portion of free water is released again under pressure with the progress of internal hydration reactions. Thus, the cement undergoes hydration reactions again at the later stage. These hydration products fill the pores between particles and reduce the proportion of cumulative pores. The pore sizes of all UHPC samples after 28 days of curing are mainly distributed around 10 nm, and with increased mixed sand replacement rate, the proportion of pores smaller than 20 nm also increases progressively, while the proportion of pores larger than 20 nm decreases.

**Figure 14:**
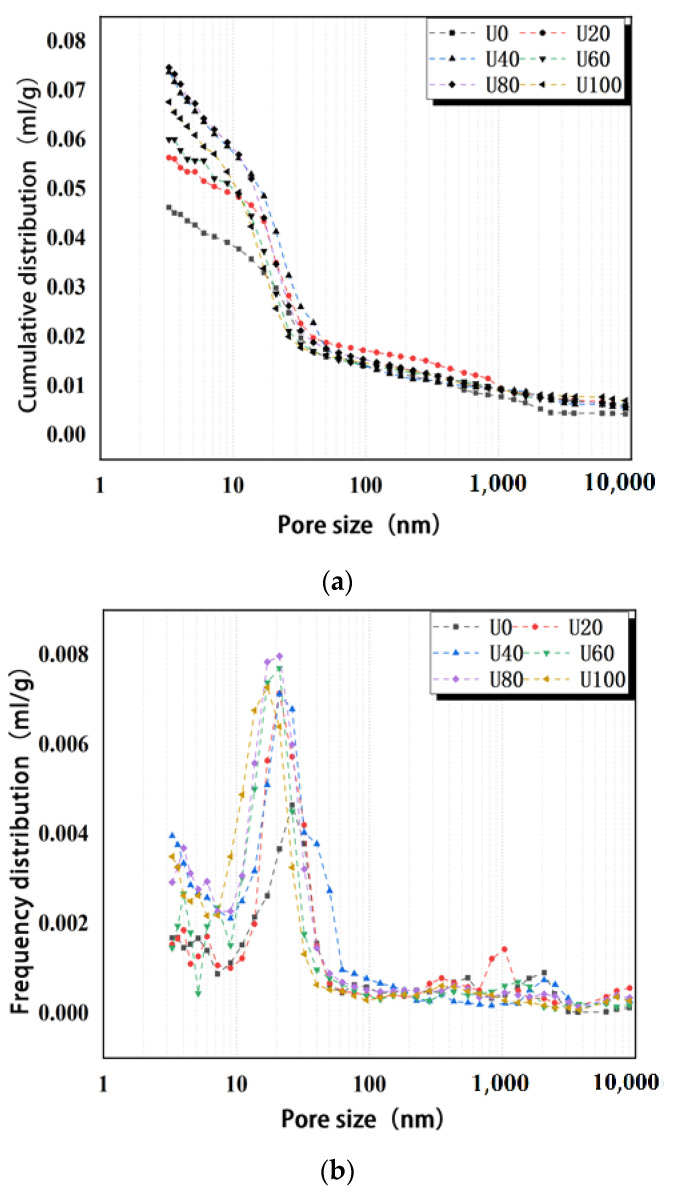

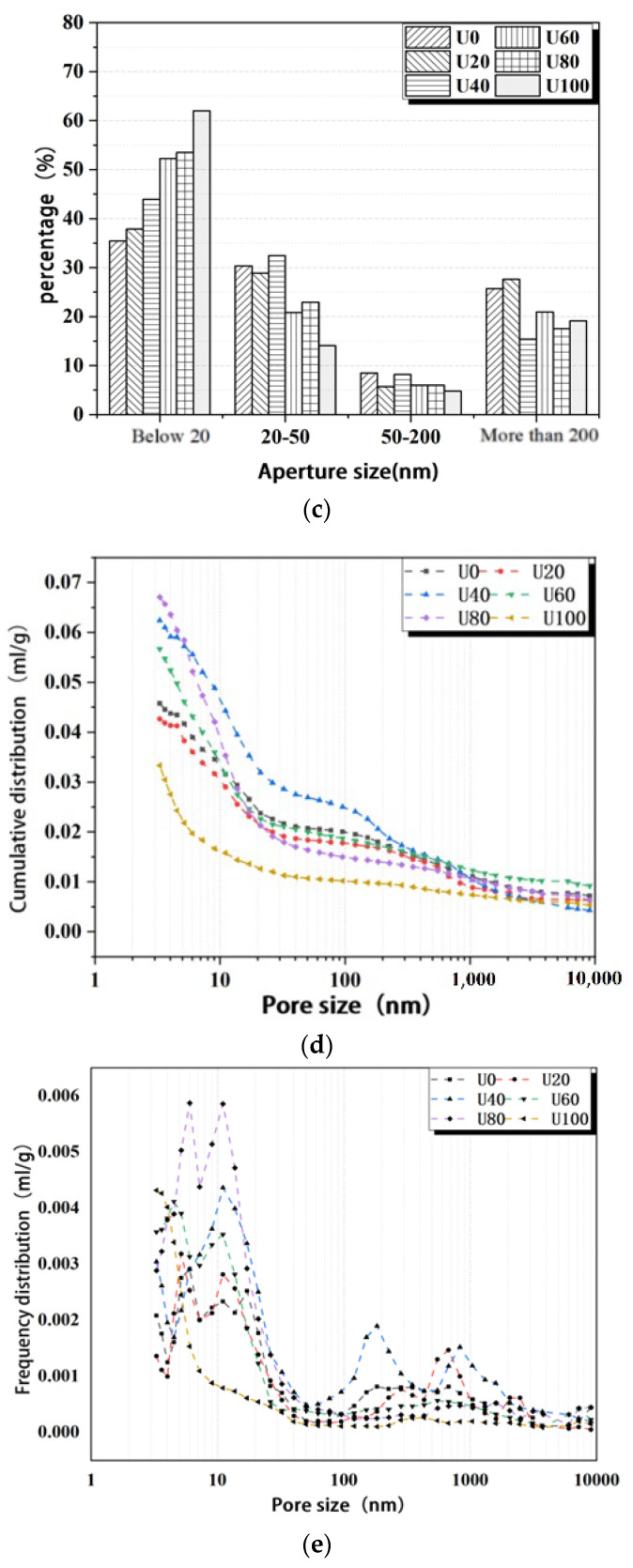

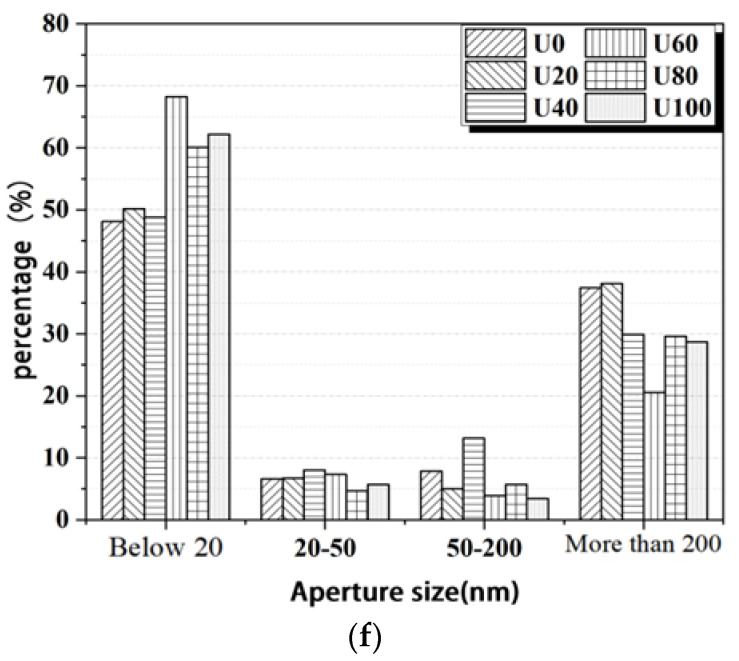
Pore structure of UHPC with different Yellow River sand content after 7 and 28 days of curing: (**a**) cumulative intrusion of 7–day test samples; (**b**) incremental intrusion of 7–day test samples; (**c**) proportion of pores of all sizes in 7–day test samples; (**d**) cumulative intrusion of 28–day test samples; (**e**) incremental intrusion of 28–day test samples; and (**f**) proportion of pores of all sizes in 28-day test samples.

The pore development of samples cured for 7 days and 28 days is compared. The results suggests that with the increase in the curing period, the proportion of cumulative pores is smaller (Figure 14) and the main distribution of pore size is smaller. When the replacement rate of mixed sand is 100%, the pore size is primarily concentrated at 3 nm, and the proportion of harmful pores decreases significantly. The addition of mixed sand leads to continuous refinement of pore size, reduces the number of harmful and multi-harmful holes in cement-based materials, facilitates the transition from harmful and multi-harmful holes to less harmful holes or even harmless holes, optimizes the pore structure, and reduces the negative effects of pore structure on strength.

##### Ecological Evaluation

**Figure 15:**
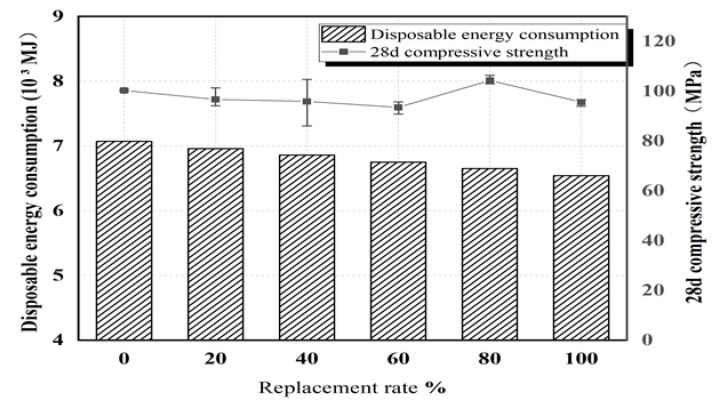
Comparison between primary energy consumption and 28-day compressive strength for various sand replacement rates.

We carried out a lifecycle ecological evaluation of Yellow River ultra-fine sand UHPC using primary energy consumption in accordance with the EN ISO 14040 standard. The ultra-fine sand of the Yellow River is natural ultra-fine sand that meets the requirements for ultra-high-performance concrete fine aggregate, while quartz sand needs to be crushed and processed, which not only wastes energy but can easily create polluting dust in the crushing process. We use production technology 1 m^3^ as revealed by the ecological evaluation of the primary energy consumed by UHPC, as presented in Figure 15. With increased substitution of river sand for quartz sand, primary energy consumption progressively decreases. When the substitution rate is 100%, primary energy consumption decreases by about 7%, with no significant decrease in compressive strength.

##### Economic Analysis

The economic analysis of this test uses the normalized cost index (NCI)—that is, the cost of raw materials per cubic meter of concrete. 

**Figure 16:**
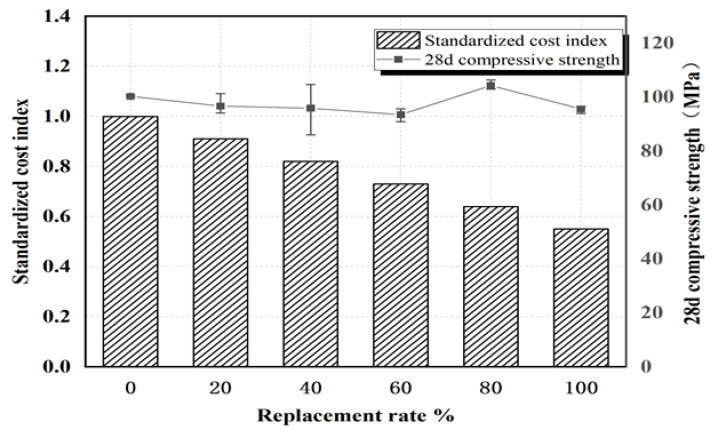
Comparison of standardized cost index and 28-day compressive strength for various sand replacement rates.

The cost declines to a certain extent with the use of mixed sand instead of quartz sand to prepare ultra-high-performance concrete. The standardized cost indexes of different replacement rates of quartz sand are determined by the algorithm of the normalized cost index. During the economic analysis, the concrete strength is also comparatively analyzed, as presented in Figure 16. As depicted in the figure, when the replacement rate of quartz sand reaches 20, 40, 60, 80, or 100%, the corresponding standardized cost indexes are 0.91, 0.82, 0.73, 0.64, and 0.55 respectively. At the 100% replacement rate, the UHPC cost decreases by 45%. Nevertheless, the strength of the ultra-fine Yellow River sand UHPC has not significantly declined, so it exhibits good value.

## 4. Results and Discussion

### 4.1. Concrete Performance

#### 4.1.1. Fluidity

Mixing superfine Yellow River sand and river sand has a negative impact on the flow performance of high-performance concrete. When the substitution rate is 20~100%, the fluidity of the corresponding mixture decreases by 0~17% and the time for the mixture to pass through a V-shaped funnel is 0.04 s~7.20 s longer than for the control group. This is because the ultra-fine sand of the Yellow River is relatively fine and has a larger specific surface area than quartz sand. These fine particles more easily absorb the water in the slurry, resulting in thickening of the slurry and decreased fluidity. However, even when the replacement rate is as high as 100%, the mixture still has high fluidity (248.5 mm).

#### 4.1.2. Mechanical Tests

Replacing quartz sand with mixed sand as aggregate does not have a serious negative impact on the compressive strength of the sample. This conclusion is also supported by the microstructure of the interface transition zone. When the replacement rate of mixed sand increases from 20, 40, 60, 80, or 100%, the compressive strength after 28 days of curing is 100.3, 96.7, 95.9, 93.5, 104.3, and 95.6 MPa, respectively, which has no obvious regularity, but the average compressive strength is 97.2 MPa. This is because the particle size distribution of mixed sand is uniform and continuous, and the ultra-fine Yellow River sand is smaller than quartz sand, which is more conducive to filling the pores between aggregates. At the same time, thanks to the modified Andreasen and Andersen model, the internal particles are densely stacked and the structural framework is dense.

### 4.2. Pore Structure and Microscopic Measurement

The addition of mixed sand increases the early shrinkage of the sample. The early shrinkage of samples with 0%, 60%, and 100% replacement rates of mixed sand is 759, 815, and 861 ppm, respectively. This is because the ultra-fine sand of the Yellow River absorbs more water during the mixing process due to its small particle size and large specific surface area. In this way, the water–binder ratio and internal humidity of the sample are reduced at the same time, increasing early shrinkage of the sample.

### 4.3. Ecological and Economic Evaluation

Replacing quartz sand with mixed sand has an adverse effect on the pore structure of early samples. However, with increased curing time, this adverse effect is offset as the pores between large particles are filled with ultra-fine Yellow River sand and with the secondary hydration reaction of cement. It is worth noting that with increased curing time and a replacement rate of mixed sand of 100%, the pore diameter is mainly distributed at 3 nm. The addition of mixed sand continuously refines the pore diameter and optimizes the pore structure. When the replacement rate of mixed sand is 100%, the cost of high-performance concrete decreases by 45% and primary energy consumption drops by 7% with no significant decrease in 28 d compressive strength.

## 5. Conclusions

In this study, the resource utilization of the sediment of the Yellow River is investigated. To reduce the limitations of existing sediment utilization schemes, we propose using sediment to build embankments and to prepare ultra-high-performance concrete (UHPC) from the ultra-fine sand of the Yellow River, and the performance of the prepared concrete is examined. The major conclusions are drawn below:

(1) Since sediment resources have been underutilized, this study proposes sediment-based embankment building, i.e., transporting sediment from the bottom of the reservoir to the shore and building an embankment at the 330 m elevation line on both sides of the reservoir. This would be consume considerable sediment and increase the water storage capacity of the reservoir area.
(2) To solve the low strength of Yellow River sand concrete, we propose a method for utilizing the ultra-fine Yellow River sand to make high-strength concrete, i.e., mixing of the ultra-fine Yellow River sand and river sand negatively affects the fluidity of ultra-high-performance concrete; however, even with a replacement rate as high as 100%, the mixture still exhibits high fluidity (248.5 mm). The addition of mixed sand does not seriously reduce the compressive strength. At a 100% replacement rate, pore diameter is primarily distributed at 3 nm. At the 100% replacement rate, UHPC cost can be reduced by 45% and primary energy consumption by 7% with no significant decrease in compressive strength.

## Figures and Tables

**Table 1:** Raw material composition.

Form	Na_2_O	MgO	Al_2_O_3_	SiO_2_	P_2_O_5_	TiO_2_	SO_3_	K_2_O	CaO	Fe_2_O_3_	LOI
Cement	0.09	1.61	4.18	19.2	0.09	-	3.35	0.78	64.93	3.32	2.49
Silica fume	0.13	0.47	0.25	94.65	0.17	-	0.69	0.84	0.36	0.15	2.29
Fly ash	0.33	0.23	38.01	46.44	0.06	-	0.69	0.88	7.5	3.12	2.79
Yellow River sand	1.65	1.88	10.79	72.73	0.17	0.83	-	3.33	3.93	4.31	0.38
Large-size river sand	1.75	1.65	11.58	75.89	0.23	0.9	-	3.21	2.29	2.3	0.2
Quartz sand	1.3	0.8	0.9	88.25	0.15	0.75	-	3.35	2.45	1.2	0.85

**Table 2:** Mix proportions of ultra-fine Yellow River sand for ultra-high-performance concrete (kg/m^3^).

Number	Cement	Fly Ash	Silica Fume	River Sand	Sand of the Yellow River	Small Quartz Sand	Large Quartz Sand	Water	Water Reducing Agent
U0	723	181	174	0	0	811	195	194	30
U20	723	181	174	44	157	649	156	194	30
U40	723	181	174	88	314	487	117	194	30
U60	723	181	174	132	472	324	78	194	30
U80	723	181	174	176	629	162	39	194	30
U100	723	181	174	220	789	0	0	194	30

U0, U20, U40, U60, U80, and U100, respectively, represent replacement rate of mixed sand to quartz sand of 0%, 20%, 40%, 60%, 80% and 100%.

## Data Availability

The data presented in this study are available on request from the corresponding authors.
